# A versatile integrated tube for rapid and visual SARS-CoV-2 detection

**DOI:** 10.3389/fmicb.2022.1070831

**Published:** 2023-01-12

**Authors:** Jingsong Xu, Xi Wang, Shuang Yang, Lei He, Yuting Wang, Jiajun Li, Qian Liu, Min Li, Hua Wang

**Affiliations:** ^1^Department of Laboratory Medicine, Renji Hospital, School of Medicine, Shanghai Jiao Tong University, Shanghai, China; ^2^Shanghai Municipal Hospital of Traditional Chinese Medicine, Shanghai University of Traditional Chinese Medicine, Shanghai, China

**Keywords:** integrated tube, SARS-CoV-2, CRISPR-Cas12a, recombinase polymerase amplification, visual detection

## Abstract

The coronavirus disease 2019 (COVID-19) caused by novel severe acute respiratory coronavirus 2 (SARS-CoV-2) has been rapidly spreading worldwide. Rapid and widespread testing is essential to promote early intervention and curb the ongoing COVID-19 pandemic. Current gold standard reverse transcription-polymerase chain reaction (RT-PCR) for detecting SARS-CoV-2 is restricted to professional laboratories and well-trained personnel, thus, limiting its widespread use in resource-limited conditions. To overcome these challenges, we developed a rapid and convenient assay using a versatile integrated tube for the rapid and visual detection of SARS-CoV-2. The reaction conditions of the method were optimized using SARS-CoV-2 RNA standards and the sensitivity and specificity were further determined. Finally, it was verified on clinical specimens. The assay was completed within 40 min, and the result was visible by the naked eye. The limits of detection (LODs) for the target ORF1ab and N genes were 50 copies/μl. No cross-reactivity was observed with the RNA standard samples of four respiratory viruses or clinical samples of common respiratory viral infections. Ninety SARS-CoV-2 positive and 30 SARS-CoV-2 negative patient specimens were analyzed. We compared these results to both prior and concurrent RT-PCR evaluations. As a result, the overall sensitivity and specificity for detection SARS-CoV-2 were 94.5 and 100.0%, respectively.

**Conclusion:**

The integrated tube assay has the potential to provide a simple, specific, sensitive, one-pot, and single-step assay for SARS-CoV-2.

## Introduction

**FIGURE 1:**
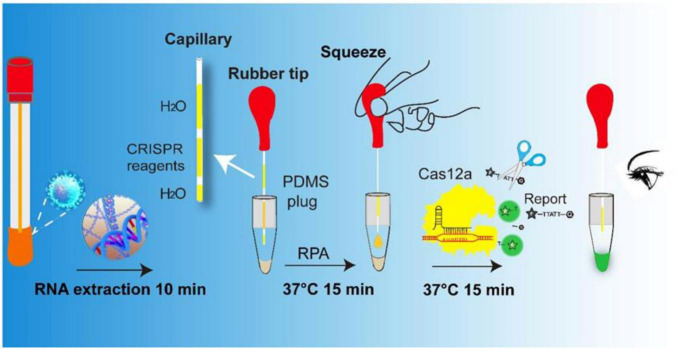
Scheme of the integrated tube assay platform for SARS-CoV-2 detection with the naked eye. Nucleic acids were extracted from the nasopharyngeal swabs (10 min) and then subjected to RT-RPA analysis at 37°C for 15 min. The corresponding RT-RPA products were verified by CRISPR-Cas12 system. The rubber tip was gently pressed and CRISPR reagents droplet was introduced into the amplified products. The reaction was conducted at 37°C for another 15 min and the results was observed by naked eye under a blue light gel imager.

The coronavirus disease 2019 (COVID-19), caused by the severe acute respiratory syndrome coronavirus 2 (SARS-CoV-2), became a worldwide pandemic and rapidly spread. The disease causes a series of non-specific symptoms such as fever, sleepiness, and cough in infected patients. However, subsequent studies have found that patients with SARS-CoV-2 exhibit high heterogeneity in their symptoms. Mild cases can be asymptomatic, while severe cases can have acute kidney injury, liver dysfunction, acute respiratory distress syndrome, and even death (Ghaebi et al., 2021; Ramos-Casals et al., 2021). This pandemic poses an unprecedented challenge to the global health system and the epidemiological statistics. To control the disease, early or asymptomatic infections must be screened and isolated in a timely manner. Rapid, highly sensitive and inexpensive laboratory tests play an important role in screening the infected patients. Currently, there are two main SARS-CoV-2 detection methods applied in clinical practice. The first is immunological diagnostic techniques represented by immunochromatography assay (ICA) (Norz et al., 2021; Zhang et al., 2021) and chemiluminescence immunoassay (CLIA) (Padoan et al., 2020). The second is molecular diagnostic technology represented by reverse transcription-polymerase chain reaction (RT-PCR). The products based on ICA are portable and fast, but lack of satisfactory specificity and sensitivity. Compared with ICA, CLIA based antigen and antibody detection have improved specificity and sensitivity and can achieve quantitative detection. However, antibody detection is suitable for population immunity investigation, but not for early diagnosis (5–7 days window) (Okba et al., 2020). In comparison to the nucleic acid amplification test, antigen tests have less sensitive (Fujiya et al., 2022). Due to its unparalleled analytic accuracy, RT-PCR remains the gold standard for molecular diagnosis of the SARS-CoV-2 (Rahbari et al., 2021). However, RT-PCR is usually restricted to professional laboratories with specialized equipment and well-trained personnel. This limits its application in some remote areas, especially in areas with inadequate medical infrastructure, as well as in secondary medical institutions in developed areas. Therefore, there is an urgent need to develop a convenient assay for the fast and early detection of SARS-CoV-2 in resource-limited conditions. Compared with RT-PCR, recombinase polymerase amplification (RPA) does not require a PCR machine, being more suitable for point-of-care testing (POCT). It is known as a nucleic acid detection technique that can replace PCR and has the advantages of rapid, high sensitivity and isothermal (Yang et al., 2018; Ivanov et al., 2021). However, since there is no thermal cycling to avoid binding between primers, it is difficult to avoid non-specific amplification in RPA reaction. Detection methods named SHERLOCK (Gootenberg et al., 2017, 2018) and DETECT (Chen et al., 2018) were successful in target detection based on CRISPR/Cas13 and Cas12a, respectively. However, this system requires an additional step of transferring the product to another test tube or test strip (Zhu et al., 2020), thereby increasing chance of aerosol contamination. Several groups have developed close-tube reactions for SARS-CoV-2 detection (Ding et al., 2020; Wang et al., 2021). However, this system requires use of high-speed centrifugation with mixed CRISPR reagents and amplification products, which is usually not feasible in field settings. In addition, when there is more CRISPR reagent on the tube lid, the reagent flows along the tube wall into the RPA reaction that is undergoing amplification, thus affecting the results. Therefore, there is an urgent need to establish a simple, fast, one-tube, and no auxiliary equipment method for the detection of the SARS-CoV-2. Here, an integrated tube, mainly made of polydimethylsiloxane (PDMS)/glass capillary and PCR tube, was constructed for visually detecting the SARS-CoV-2. As shown in Figure 1, the assay is mainly divided into the following steps: extraction of nucleic acid, RPA pre-amplification, Cas12a recognize the target sequence and cleavage of the ssDNA reporter, and visual SARS-CoV-2 detection. Unlike previously reported CRISPR based assays, the Cas12a reagents were sealed in the PDMS/glass capillary tube and the reaction between Cas12a and RPA amplification product can be performed through simple manual operation without centrifuge and other auxiliary equipment in this assay. As shown, SARS-CoV-2 RNA templates extracted from the nasopharyngeal swab samples were amplified by RT-RPA, followed by mixing with the Cas12a reagents for cleavage using the rubber tip. In this work, the quenched fluorescent single-stranded DNA reporter (FAM-TTATT-BHQ1) was introduced. Once the Cas12a protein was activated by a crRNA recognized DNA target, it splits the quenched fluorescent ssDNA reporter indiscriminately, generating the fluorescence signal visible to the naked eye under blue light (470 nm). It provides a rapid, inexpensive, and convenient assay for SARS-CoV-2 nucleic acid detection.

## Materials and methods

### Materials

The RNA standards for SARS-CoV-2, SARS-CoV, Parainfluenza virus, Influenza virus A (FluA), and Influenza virus A (FluB) were synthesized by the Shanghai Institute of Measurement and Testing Technology. All primers, CRISPR RNA (crRNA), and the ssDNA reporter (FAM-TTATT-BHQ1) were synthesized by the Sangon Biotech Co. Ltd., Shanghai, China. EnGen^®^ Lba Cas12a was purchased from New England Biolabs (Ipswich, MA, UK). The RNase inhibitor was purchased from Takara and the RPA amplification kit was purchased from Leshang Biotechnology (Wuxi) Co., LTD., China.

### PDMS/glass capillary fabrication

**FIGURE 2:**
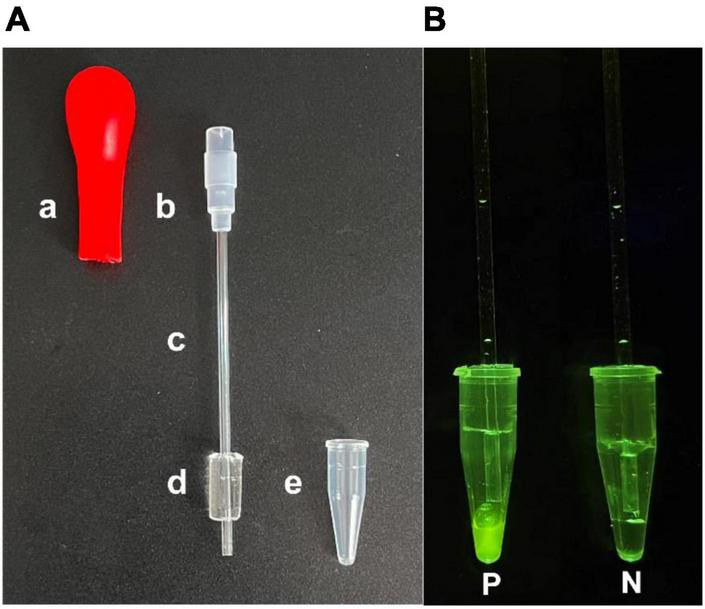
The versatile integrated tube. The integrated tube consisted of five parts **(A)**, namely (a) rubber tip, (b) joint, (c) glass capillary, (d) PDMS plug and (e) PCR tube. Integrated tube based visual assay results **(B)**: P, positive; N, negative.

As shown in Figure 2A, the integrated tube consisted of five parts, namely (a) rubber tip, (b) joint, (c) glass capillary (internal diameter 42.5 μm; height 70 mm, volume 40 μl), (d) PDMS/Glass capillary and (e) PCR tube (volume 300 μl). PDMS/glass capillary was produced by molding a PDMS silicone elastomer against a hollow polypropylene mold (diameter 6 mm; height 9 mm). The PDMS precursor mixture, prepared at a weight ratio of base to curing agent of 10:1, was poured carefully in a plastic cup and mixed by gentle stirring, and then placed under a vacuum for 0.5 h to remove the bubbles. The glass capillary was passed through polypropylene mold and secured with a plastic cap. Next, the PDMS precursor mixture was slowly added into the mold. After curing at 65°C for 2 h, the mold and plastic cap was taken out from the PDMS precursor mixture. Next, PDMS/Glass capillary was combined with PCR tube to achieve rapid detection of the SARS-CoV-2. Figure 2B were the visual detection results.

### Clinical samples

One hundred and twenty nasopharyngeal swab samples were collected from Renji Hospital. Nucleic acid extraction and purification were performed using a commercial automatic nucleic acid extraction instrument (Biogerm Medical Technology Co., Ltd., BG-Nege-96, Shanghai, China) according to the manufacturer’s instructions. According to RT-PCR testing results, 90 nasopharyngeal swabs were positive, and 30 nasopharyngeal swabs were negative for SARS-CoV-2. Among those 30 SARS-CoV-2 negative samples, 2 cases were diagnosed with influenza A, 5 cases with influenza B, and 2 with respiratory syncytial virus. The current study was approved by the Ethics Committees of Shanghai Jiao Tong University School of Medicine Affiliated Renji Hospital (Approval number: KY2022-192).

### Primers and crRNAs design

The RPA primers were manually designed and primer sequences were summarized in Table 1. Specially, endogenous ribonuclease P RNA (RNase P) was set up as an internal reference control to monitor the process of clinical sample collection, preservation, and transportation, as well as nucleic acid extraction to avoid false negative results misinterpretation. The crRNAs were manually designed and the information of crRNAs was summarized in Table 2.

**TABLE 1:** Recombinase polymerase amplification (RPA) primer set design.

Primer name	Sequence (5′ to 3′)
ORF1ab-F1	TCTGTAGTACTATGACCAATAGACAGTTTC T
ORF1ab-F2	CACTAGAGGAGCTACTGTAGTAATTGGAAC
ORF1ab-F3	CTACTGTAGTAATTGGAACAAGCAAATTCTATG
ORF1ab-R1	CACACATGACCATTTCACTCAATACTTGAGC
ORF1ab-R2	CACTCAATACTTGAGCACACTCATTAGCTAATC
ORF1ab-R3	CTATAGAAACGGTGTGACAACCTACAACACG
N-F1	GATCACATTGGCACCCGCAATCCTGCTAAC
N-F2	CAATGCTGCAATCGTGCTACAACTTCCTC
N-F3	CTACAACTTCCTCAAGGAACAACATTGCCA
N-R1	GCCTTGTTGTTGTTGGCCTTTACCAGACAT
N-R2	CTCTCAAGCTGGTTCAATCTGTCAAGCAGCA
N-R3	CAAGCAGCAGCAAGCAAGAGCAGCATCAC
RNaseP-F	GCCTGGAGGGCCCTGTGGAACGAA
RNaseP-R	TTCTGGCAGCGGGCCAGCTGGGCCTTAA

**TABLE 2:** Details of crRNAs of the SARS-CoV-2 and RNaseP.

crRNA name	Sequence (5′ to 3′)
N gene	GAAUUUCUACUGUUGUAGAUUUGAACUGUUGCGACUACGUGAUG
ORF1ab gene	GAAUUUCUACUGUUGUAGAUCGAGCAAGAACAAGUGAGGCCAUA
RNaseP gene	GUGUGACCCUGAAGACUCGGUUUUAGCCACUGACUCGGAUC

### Single-step and one-pot visual detection of SARS-CoV-2

For one-pot visual detection, 5 μl of RT-RPA reaction mixture including 1 μl of RNA standards or 1 μl of RNA from clinical samples was added at the bottom of PCR tube.

Five microliters of sterile water, 15 μl of Cas12a reaction solution, and 5 μl of sterile water were added sequentially to the capillary tubes. The Cas12a reaction solution contained 1.2 μM crRNA, 0.4 μM Cas12a, 2 μM ssDNA reporter, 12 U RNase inhibitor.

The PDMS/glass capillary was inserted into PCR tube. After 15 min of reaction at 37°C, the rubber tip was gently pressed and Cas12a reaction droplet was introduced into the PRA amplified products. Then, Cas12a reaction was conducted at 37°C for another 15 min and green fluorescent signals could be observed by naked eye under a blue light gel imager.

### Fluorescence detection

To further validate the visual detection accuracy, the signals of the method were quantified using a fluorescence detection system. Briefly, 1 μl of the RPA reaction products were added to the plate and then the fluorescence intensity was determined on a fluorescence microplate reader synergy H1 (Biotek Instruments, Winooski, Vermont, USA) using excitation at 470 nm and emission at 510 nm. In each replicate, fluorescence intensities of CRISPR-Cas12 system reactions with various detection concentrations were normalized against the one containing the highest target concentration. The cutoff value was confirmed after calculating the experimental results of negative and positive controls and observing by naked eyes. In fact, the cutoff number 4 is a logarithmic value. When the raw fluorescence intensity is higher than 10,000, the number will be bigger than 4 after being calculated. In our research, the fluorescence intensity of all the positive controls was higher than 10,000, while those of the negative controls were around 500, which showed a very significant difference. Meanwhile, obvious fluorescence can also be observed by naked eye when fluorescence intensity was higher than 10,000. For expression purposes, we choose the logarithmic value 4 as the cutoff value.

### Real-time RT-PCR

RNA was extracted from 300 μl nasopharyngeal swab samples in a biological safety cabinet using the Bio-germ nucleic acid extraction and purification Kits. Real-time RT-PCR was conducted for comparison with our method. Real-time RT-PCR assays of N and ORF1ab genes of SARS-CoV-2 were used in accordance with the respective manufacturers’ instructions (2019-nCoV detection kits from fluorescence PCR, Shanghai Bio-germ medical science and Technology Co., Ltd, China). Reactions were conducted in 25 μl volumes in an ABI 7500 real-time PCR system using 5 μl of nucleic acid. Usually, the reaction cycle parameters were set as reverse transcription at 50°C for 10 min, denaturation at 95°C for 5 min, then followed by 45 two-step cycles of 10 s at 95°C and 40 s at 55°C.

### Statistical analysis

Statistical significance was calculated by GraphPad Prism (Vision 8.0.2) and all the data were shown as mean ± s.d. All illustrations were drawn by GraphPad Prism. The bar chart and line chart were drawn with column and group respectively. The heat map was initially drawn by Graphpad Prism and adjusted by Adobe Illustrator. Two-tailed Mann–whitey U test was used to compare two groups with a *P*-value < 0.05 as a threshold for significance. Three technical replicates were performed to improve the statistics. Pearson correlation coefficient was used to analyze the correlation.

## Results

### Development and optimization of the reaction system

**FIGURE 3:**
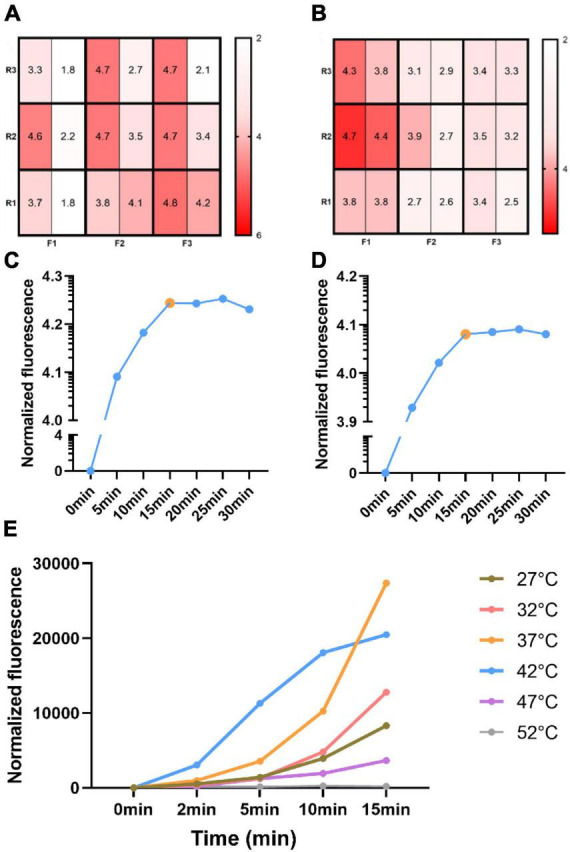
Development and optimization of the reaction system. Heat maps of RPA primer screening of ORF1ab gene **(A)** and N gene **(B)**. The abscissa represents three pairs of forward primers and the ordinate represents three pairs of reverse primers. In each box, the left side represents the result under the high concentration template and the right side represents the result under the low concentration template. A time-dependent curve of the RPA reaction **(C)**. The orange dot represents the time of the arrival of the platform stage. A time-dependent curve of the CRISPR reaction **(D)**. The time-dependent curves of the reaction under different temperatures **(E)**.

In this work, we first optimized the RPA assay system. Three front primers and three reverse primers were designed according to the RPA primer design principle, 9 pairs of primers were detected and then selected one pair of primers with the highest amplification efficiency. We first employed both RPA and CRISPR-Cas12 reactions for 30 min at 37°C. High and low concentrations of template (1.0 × 10^6^ copies/μl, 1.0 × 10^4^ copies/μl) were employed and the fluorescence intensities were measured to reflect the amplification efficiency of each primer combination. The results of the RPA primer combination assay for ORF1ab gene and N gene were shown in Figures 3A, B, respectively. The abscissa and the ordinate represent the forward primers and the reverse primers, respectively. In each box, the left side represents the result under the high concentration template and the right side represents the result under the low concentration template. The fluorescence intensity of primer combination (F3R1) was significantly higher than the cutoff value (Figure 3A), and therefore, this primer pair was used in subsequent experiments. We performed an RPA primer screening test for the N gene using the same reaction conditions. The amplification efficiency of primer combination (F1R2) was optimal at both high and low template concentrations and exceeded the cutoff value of 4.0 (Figure 3B). Therefore, F1R2 was used in subsequent experiments.

Then, to achieve better reaction sensitivity and efficiency, we tested the effect of different reaction conditions on the results. Since the RPA used in the reaction system had the best activity at 37°C, we focused on this study by timing of RPA amplification. The fluorescence intensity was examined after RPA reaction at 37°C for 0, 5, 10, 15, 20, 25, and 30 min, respectively. It was easy to find that the obvious fluorescence can also be seen by naked eye and the normalized fluorescence intensity has exceeded the cutoff value of 4.0 at 5 min (Figure 3C). However, the fluorescence intensity remained essentially unchanged after 15 min, which meant the reaction entered a plateau period. Therefore, we chose 15 min as the optimal time for subsequent RPA reactions.

Next, the reaction time of the CRISPR reaction was optimized. The CRISPR-Cas12a reactions were performed using the same amplicon samples at 37°C for 0, 5, 10, 15, 20, 25, and 30 min. The time-dependent curves of the reaction were shown in Figure 3D. The fluorescence intensity increased with time significantly from 0 to 15 min, but it did not change with time after 15 min. Therefore, we chose 15 min as the reaction time for subsequent CRISPR-Cas12a reaction, which is significantly shorter than the previous studies (Swarts et al., 2017).

Finally, the optimal temperature of the method was explored at different temperatures (from 27 to 52°C) for 15 min. As shown in Figure 3E, the fluorescence intensity at 37°C was the highest at 15 min. Therefore, 37°C was used as the optimal reaction temperature for subsequent studies.

### Limit of detection and specificity of the reaction system

**FIGURE 4:**
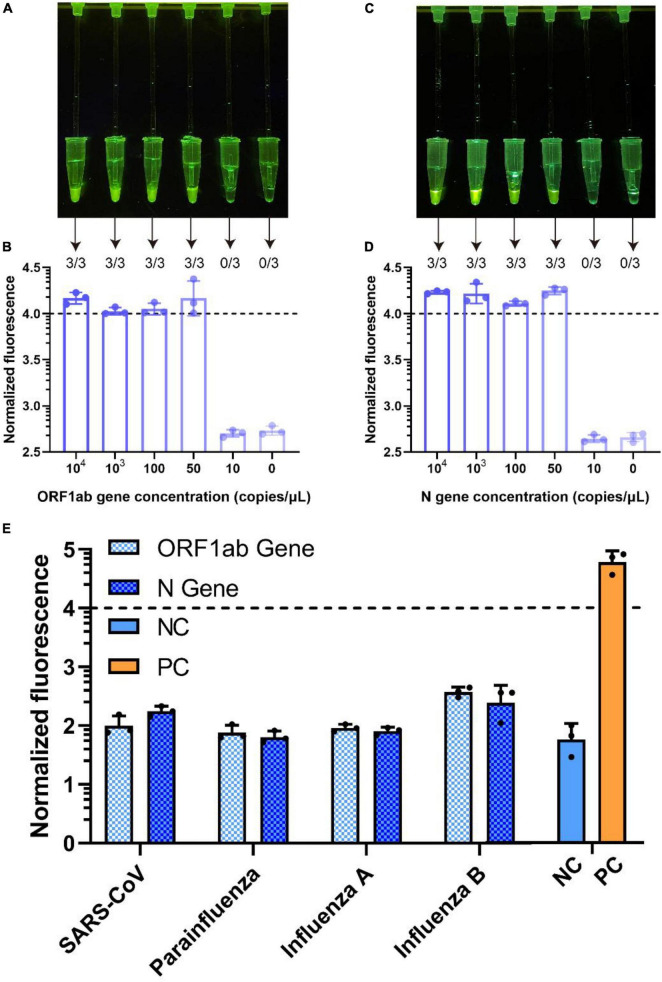
Limit of detection and specificity of the reaction system. The LOD of the ORF1ab gene [**(A)**, visual detection; **(B)**, fluorescence detection]. The LOD of the N gene [**(C)**, visual detection; **(D)**, fluorescence detection]. All examinations were performed in triplicate. Four pathogen standards were used to detect the specificity of the integrated assay **(E)**. The orange line is the positive control, which is the only one above cutoff value (4.0). The fluorescence intensity of the reaction was normalized, bar represent mean ± s.d.

After the visualized reaction system was established and optimized, we used standard substances to determine the limit of detection (LOD) and specificity of the system. To identify the LOD of the assay, SARS-CoV-2 standard substances were diluted (1.0 × 10^4^, 1.0 × 10^3^, 1.0 × 10^2^, 50, 1.0 × 10^1^ copies/μl). All examinations were performed in triplicate. We first quantified the fluorescence of the reaction products, after which we set the normalized fluorescence cutoff value of the detection at 4.0. As shown in Figure 4, the normalized fluorescence was above 4.0 and obvious fluorescence could be seen by naked eye. The results showed that the LODs for ORF1ab gene and N gene were 50 copies/μl (Figures 4A, B) and 50 copies/μl (Figures 4C, D), respectively.

Next, to demonstrate the specificity of the assay, we selected four standards as targets for the specificity assay, including SARS-CoV, parainfluenza virus, influenza A virus and influenza B virus. The results were detected by naked eye and the fluorescence detection system. As shown in Figure 4E the detection results of the four standard samples in the systems were all negative, the normalized fluorescence was below the cutoff value and no fluorescence was observed by naked eye. Therefore, the assay was highly specific, without obvious cross-reactivity with other respiratory viruses.

### Clinical validation of the optimized reaction system

**FIGURE 5:**
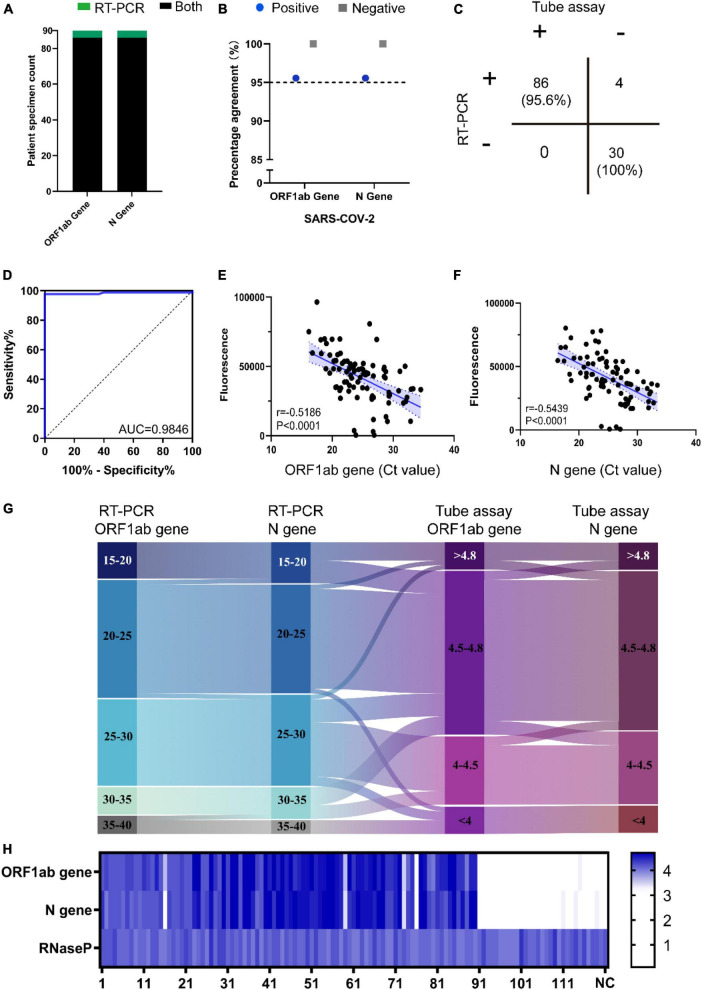
Clinical validation of the optimized reaction system. **(A)** Comparison of the performance of RT-PCR and the visualized method on 90 SARS-CoV-2 patient specimens. Black: genes correctly identified by both Integrated tube assay and RT-PCR. Green: RT-PCR only. **(B)** Positive and negative percentage agreement for genes on the assay. Blue: positive; Black: negative. **(C)** Concordance of the performance of RT-PCR to this assay for 120 clinical samples. **(D)** The receiver operating curve (ROC) analysis. **(E)** Pearson correlation analysis was used to analyze the correlation between the Ct values of ORF1ab gene and the fluorescent intensity values. **(F)** Pearson correlation analysis was used to analyze the correlation between the Ct values of N gene and the fluorescent intensity values. **(G)** The sankey diagram showing the correlation between the range of Ct values and the fluorescent intensity values. **(H)** Normalized fluorescence signal for each SARS-CoV-2 positive sample on the reaction system.

Compared with the standard substances, there are a variety of interfering substances in clinical samples, including human-derived substances, contamination during sampling and other pathogens (Xu et al., 2020). Next, the assay was further tested using 120 clinical samples. Ninety SARS-CoV-2 positive samples and 30 negative samples were recruited. Among those SARS-CoV-2 positive samples, 86 cases ORF1ab and N cases genes were detected by the assay (Figure 5A). However, we failed to test both genes in four cases, No.16, No.59, No.73, and No.76 (Figure 5E). In consistent with the RT-PCR results, none of the genes for SARS-CoV-2 were detected among those 30 SARS-CoV-2 negative samples, including influenza A (2 cases), influenza B (5 cases), and respiratory syncytial virus (2 cases) (Figure 5B). The results shown that the negative predictive value was 100% and the positive predictive value was 95.6% (Figure 5C). To determine the sensitivity and specificity of the assay, we performed a receiver operating curve (ROC) analysis. The results shown that the sensitivity of the assay was 94.5% and the specificity was 100% (Figure 5D). Next, pearson correlation coefficients were used to evaluate the relationships between the Ct values and the fluorescent intensity values. The Ct values of ORF1ab gene showed a negative correlation with the fluorescent intensity values (*r* = −0.5186, *P* < 0.0001) (Figure 5E). Similar trends were detected at the Ct values of N gene, which is negatively correlated with the fluorescent intensity values (*r* = −0.5439, *P* < 0.0001) (Figure 5F). The correlation between the range of Ct values and the relative fluorescence values was visualized as a Sankey diagram in Figure 5G. The fluorescence intensity of the results was shown in Figure 5H. In summary, the reaction system had high specificity, good anti-interference ability and stability, so it was suitable for genetic testing in clinical practice.

## Discussion

In this study, we have developed an intergrade tube assay for rapid and visual SARS-CoV-2 detection. The visual detection process could be completed within 40 min. The LOD of ORF1ab gene and N gene of SARS-CoV-2 were 50 copies, respectively. We first set up a method of screening RPA primers, which was using high and low concentration templates to detect the fluorescence intensity of different primers and selected the primer combination with the highest amplification efficiency after considering the results of two experiments. Then we optimized the reaction conditions, including RPA reaction time, the CRISPR reaction time, and CRISPR reaction temperature. The reaction conditions were finally determined to be isothermal at 37°C for 15 min. Compared with RT-PCR, the method used in this study did not require specific, expensive, and sophisticated equipment. A simple water bath or heat block is sufficient to provide a constant temperature for reactions. In addition, the detection time was significantly shorter than conventional RT-PCR. The greatest advantage of the method was the visual interpretation by naked eye without any testing equipment for positive and negative results. The results showed that the LODs of the visualized method for ORF1ab and N genes were 50 copies/μl. In terms of detection specificity, there was no cross reaction with SARS-CoV, parainfluenza virus, influenza A virus and influenza B virus, which was very helpful in distinguishing this group of clinically similar human respiratory pathogens. The clinical symptoms caused by these viruses were very similar to those after SARS-CoV-2 infection and were sometimes difficult to identify (Coutinho et al., 2020; Lai et al., 2020; Wu et al., 2020). The sequence homology of coronavirus and SARS-CoV-2 also lead to misdiagnose of the two viruses by some non-specific method such as antigen detection (Lai and Lam, 2021).

The detection of clinical samples confirmed the anti-interference ability, specificity and robustness of the reaction system. The method tested 30 SARS-CoV-2 negative samples with a variety of common respiratory pathogens and did not interfere in any samples.

The detection rate of 90 positive samples was 95.6%. Compared with PCR, the method is relatively sensitive and could only detect clinical samples with a Ct value of 35. Therefore, the new method can only be used for preliminary screening of the SARS-CoV-2. Application of the new method has the potential to rapidly screen out asymptomatic SARS-CoV-2-infected cases and thereby curb further spread of the virus.

RT-PCR is still the gold standard for the diagnosis of SARS-CoV-2 (Gao and Quan, 2020; Layden et al., 2020). However, this method is demanding for testing sites and personnel. Compared with PCR, this method is simple, and rapid, does not require specialized laboratories and expensive instruments, and is therefore more suitable for widespread development in primary care settings. Antigen detection provides the possibility of home detection for potentially infected patients, but its sensitivity and specificity cannot meet the diagnostic requirements (Lambert-Niclot et al., 2020). Thereby, this work provides a rapid, inexpensive, and convenient assay for SARS-CoV-2 nucleic acid detection and is more easily developed for point of care testing applications. In the future, nucleic acid extraction will be integrated into the tube and more easily developed for point of care testing applications.

## Conclusion

In summary, we have developed an assay for SARS-CoV-2 detection. The visual detection process could be completed within 40 min. The LOD of ORF1ab gene and N gene of SARS-CoV-2 were 50 copies, respectively. This simple, fast, high-specific, and convenient method can be a suitable tool to screen SARS-CoV-2 in hospital emergency.

## Data availability statement

The original contributions presented in this study are included in the article/supplementary material, further inquiries can be directed to the corresponding authors.

## Ethics statement

This current study was approved by the Ethics Committees of Shanghai Jiao Tong University School of Medicine Affiliated Renji Hospital (Approval number: KY2021-192). The patients/participants provided their written informed consent to participate in this study.

## Author contributions

HW and ML designed the study. JX, XW, LH, and YW performed the experiments. JX analyzed the data and wrote the discussion part of the manuscript. JL and QL wrote the “Results” section of the manuscript. All authors reviewed the manuscript.
